# What are the physical and mental health implications of duration of untreated psychosis?

**DOI:** 10.1192/j.eurpsy.2021.22

**Published:** 2021-03-29

**Authors:** Gonzalo Salazar de Pablo, Daniel Guinart, Christoph U. Correll

**Affiliations:** 1 Early Psychosis: Interventions and Clinical-detection (EPIC) lab, Department of Psychosis Studies, Institute of Psychiatry, Psychology & Neuroscience, King’s College London, London, UK; 2 Institute of Psychiatry and Mental Health, Department of Child and Adolescent Psychiatry, Hospital General Universitario Gregorio Marañón, School of Medicine, Universidad Complutense, Instituto de Investigación Sanitaria Gregorio Marañón (IiSGM), CIBERSAM, Madrid, Spain; 3 The Zucker Hillside Hospital, Department of Psychiatry, Northwell Health, Department of Psychiatry, Northwell Health, Glen Oaks, NY, USA; 4 Department of Psychiatry and Molecular Medicine, Donald and Barbara Zucker School of Medicine at Hofstra/Northwell, Hempstead, NY, USA; 5 Center for Psychiatric Neuroscience, The Feinstein Institutes for Medical Research, Manhasset, NY, USA; 6 Department of Child and Adolescent Psychiatry, Charité Universitätsmedizin, Berlin, Germany

Psychotic disorders, such as schizophrenia, are among the most severe and debilitating conditions in psychiatry, and in all of medicine. Duration of untreated psychosis (DUP) is the period that goes from the onset of psychosis to the start of an adequate treatment. DUP is an important prognostic factor, as longer DUP has been associated with poor mental health outcomes, including more severe positive and negative symptoms and decreased likelihood of remission [1]. However, DUP has been a source of controversy lately, particularly regarding its relationship with psychosocial functioning, arguably one of the most important mental health outcomes for psychiatric patients along with quality of life. While a longer duration of psychosis has been consistently associated with poor psychosocial functioning, the importance of this initial period until individuals with psychosis get an appropriate treatment (i.e., DUP) has been a subject of discussion. Two main opposed scenarios have been presented:

According to the first scenario, the decline in functioning is a part of the disorder’s natural history, and while in some psychotic patients, this decline occurs prior to the first hospitalization, in others, this is observed after the first admission, due to earlier detection [2]. The observation of similar declines in psychosocial functioning after 20 years in individuals from a single cohort, regardless of length of DUP [2], has given rise to the hypothesis that lead-time bias (LTB) may confound the association between longer DUP and poorer psychosocial functioning in schizophrenia. LTB refers to the latency between the time in which an early diagnosis is established and the time in which a disorder would have been diagnosed without early detection or screening, which artificially increases the time before a poor outcome occurs. LTB has been observed in cancer research [3], affecting such essential outcome as mortality. According to this scenario, efforts to carry out early detection and early intervention strategies would be mostly futile, which questions the need of primary and secondary prevention in the psychotic disorders field. Notably, within this scenario, the presence of LTB—as it happens in cancer research—does not necessarily explain all the variability between individuals who are identified promptly and those who are not. In fact, cancer research shows that an early detection and intervention in conditions in which LTB is observed may still be valuable.

The second scenario, more supported by the scientific evidence established to date, supports that prolonged DUP leads per se to a significant neurological and psychosocial damage that worsens the illness course of psychotic disorders, including psychosocial functioning. In several physical conditions, delayed detection and treatment have also been associated with severe consequences. In fact, the dramatic impact that a delayed intervention has had on some physical illnesses has led to the establishment of several preventive screening strategies in the general population—usually referred to as universal prevention. Some of these universal prevention programs have been conducted from the first stages of life, even from birth, such as the screening for phenylketonuria and congenital hypothyroidism in newborns by neonatal heel prick. Without adequate and early treatment, these conditions may be permanent and require lifelong treatment. Mammograms for women over 50 years old and pap smears in women who use oral contraceptives intend to improve the prognosis in high-risk individuals according to certain risk factors—selective prevention. Finally, clinical care for individuals with subthreshold or minimal symptoms who are at clinical high-risk for psychosis is provided in the prevention of psychosis services [4]—indicated prevention. According to this scenario and line of argument, and following this rationale, the benefits of a prompt detection and intervention—as well as preventive efforts—have meaningful and positive effects. As an example, in this field, a DUP of 4 weeks predicted >20% more severe symptoms than a DUP of 1 week according to a recent umbrella review [1].

In this discussion about the relevance and effects of DUP, several factors have clinical and practical importance. Most studies supporting either the first or the second scenario are observational, which limits inferences on causation. Although some naturalistic and observational studies have provided valuable evidence, which has advanced knowledge in the field, only clinical trials—particularly randomized clinical trials—have the optimal design to test causal relationships, answering the question of whether or not/to what degree DUP has a significant impact on functioning and other outcomes. Importantly, in randomized clinical trials, early intervention has been associated with an improvement in several mental health outcomes, including treatment discontinuation, hospitalization, and positive and negative symptom severity compared to treatment as usual [5]. These results support that prolonged DUP is associated with poor mental health outcomes, as it prevents or delays the access of these patients to specific early intervention services.

The relevance of reducing/delaying mortality as an ultimate goal for physical health and mental health conditions is undeniable. Furthermore, all interventions (preventive, early or not) should ultimately intend to ensure greater survival rates in the individuals they target. Dramatically, a 3.5-fold—and growing—increase in the mortality risk in people with schizophrenia has been observed [6]. The main reason is that individuals with schizophrenia suffer more frequently than individuals in the general population from cardiovascular morbidity and mortality [7]. The early phase of psychosis is considered a critical period for acquiring and preventing cardiometabolic risk. By systematically monitoring weight, cardiometabolic risk factors and smoking, and establishing early intervention strategies that improve physical health outcomes in these patients, this increased cardiovascular morbidity and mortality risk can be substantially reduced.

Furthermore, as metabolic syndrome, diabetes and hypertension have been associated with worse cognition in people with schizophrenia [8], shortening the duration of untreated metabolic problems and preventing or reducing cardiovascular risk factor development is crucial both for physical as well as mental health outcomes. One of the factors that has an important influence on cardiometabolic risk in individuals with psychotic disorders is antipsychotic medication. In patients with schizophrenia, antipsychotics are associated with varying degrees of metabolic disturbances, but continued antipsychotic treatment has been associated with reduced all-cause mortality and, even, cardiometabolic-related mortality compared to untreated people with schizophrenia [9]. Furthermore, the use of preventive cardioprotective medications among patients with schizophrenia who had suffered cardiac events is associated with decreased cardiovascular mortality [10]. These results underscore the benefits of early (or preventive), continued and comprehensive physical and mental health care in people with psychotic disorders.

In conclusion, although we believe the hypothesis of LTB confounding the association between DUP and poor outcomes merits further study, clinicians must ensure that prevention and early intervention targeting both mental and physical health remain at the frontline of their efforts when treating patients with psychotic disorders. If subsequent clinical trials confirm the LTB hypothesis, it is likely to explain only partially the association between longer DUP and poor psychiatric and functional outcomes in individuals with psychosis. Early intervention services reducing DUP have not only the potential to improve poor mental health but also physical health outcomes, including the reduction of mortality in individuals with psychotic disorders. This improvement may be achieved through integrated care that simultaneously targets physical, particularly, cardiometabolic, and mental health outcomes, optimizing antipsychotic benefit-to-risk ratios and combining pharmacologic and lifestyle as well as psychosocial interventions early on in the illness.

## References

[1] Howes O, Whitehurst T, Shatalina E, Townsend L, Onwordi E, Mak A, et al. The clinical significance of duration of untreated psychosis: an umbrella review and random-effects meta-analysis. World Psychiatry. 2021;20:75–95. 33432766 10.1002/wps.20822 PMC7801839

[2] Jonas KG, Fochtmann LJ, Perlman G, Tian Y, Kane JM, Bromet EJ, et al. Lead-time bias confounds association between duration of untreated psychosis and illness course in schizophrenia. Am J Psychiatry. 2020;177:327–34. 32046533 10.1176/appi.ajp.2019.19030324 PMC10754034

[3] Nair HS, Knight SR, McKenzie C, MacDonald AJ, Macdonald A. Age at death and the effect of lead-time bias in patients with colorectal cancer: a 10-year follow-up. Colorectal Dis. 2019;21:775–81. 30848537 10.1111/codi.14602

[4] Salazar de Pablo G, Estradé A, Cutroni M, Andlauer O, Fusar-Poli P. Establishing a clinical service to prevent psychosis: what, how and when? Systematic review. Transl Psychiatry. 2021;11:43. 33441556 10.1038/s41398-020-01165-x PMC7807021

[5] Correll CU, Galling B, Pawar A, Krivko A, Bonetto C, Ruggeri M, et al. Comparison of early intervention services vs treatment as usual for early-phase psychosis: a systematic review, meta-analysis, and meta-regression. JAMA Psychiatry. 2018;75:555–65. 29800949 10.1001/jamapsychiatry.2018.0623 PMC6137532

[6] Olfson M, Gerhard T, Huang C, Crystal S, Stroup TS. Premature mortality among adults with schizophrenia in the United States. JAMA Psychiatry. 2015;72:1172–81. 26509694 10.1001/jamapsychiatry.2015.1737

[7] Correll CU, Solmi M, Veronese N, Bortolato B, Rosson S, Santonastaso P, et al. Prevalence, incidence and mortality from cardiovascular disease in patients with pooled and specific severe mental illness: a large-scale meta-analysis of 3,211,768 patients and 113,383,368 controls. World Psychiatry. 2017;16:163–80. 28498599 10.1002/wps.20420 PMC5428179

[8] Hagi K, Nosaka T, Dickinson D, Lindenmayer JP, Lee J, Friedman J, et al. Association between cardiovascular risk factors and cognitive impairment in people with schizophrenia: a systematic review and meta-analysis. JAMA Psychiatry. 2021. Mar 3; e210015 doi:10.1001/jamapsychiatry.2021.0015. PMC7931134 33656533

[9] Taipale H, Tanskanen A, Mehtälä J, Vattulainen P, Correll CU, Tiihonen J. 20-year follow-up study of physical morbidity and mortality in relationship to antipsychotic treatment in a nationwide cohort of 62,250 patients with schizophrenia (FIN20). World Psychiatry. 2020;19:61–8. 31922669 10.1002/wps.20699 PMC6953552

[10] Kugathasan P, Horsdal HT, Aagaard J, Jensen SE, Laursen TM, Nielsen RE. Association of secondary preventive cardiovascular treatment after myocardial infarction with mortality among patients with schizophrenia. JAMA Psychiatry. 2018;75:1234–40. 30422158 10.1001/jamapsychiatry.2018.2742 PMC6583028

